# Dentistry in Foreign Countries

**Published:** 1885-05

**Authors:** Charles A. Kingsbury

**Affiliations:** Philadelphia, Pa.


ARTICLE IV.

### DENTISTRY IN FOREIGN COUNTRIES.

BY DR. CHARLES A. KINGSBURY, PHILADELPHIA, PA.

Some of my own impressions and experiences, while travelling through Europe and the East, some years ago, may be of interest. My visits to the native dentists, and the acquaintance I formed with them, though necessarily limited, convinced me at that time that they were, in many respects pertaining to their profession, considerably below the standard of the reputable and experienced dentists of our own country. I will mention a case in point that occured during my visit to Beyrut, Syria. Beyrut contains a population of some six thousand, a large proportion being English, French and German. Soon after my arrival there, I was called on by a Turkish gentleman connected with the American Mission and College, who wished to consult with me in regard to his teeth. I found that he was wearing a full artifical denture of rubber, and that it caused him great trouble and suffering. It was obvious that the defects were so radical as to require an entire reconstruction of the dentures both upper and under. Upon stating this fact to him, he desired me to take the dentures for him. I replied that I was travelling, not professionally, but for my health and pleasure; that I had no dental instruments with me, and that it would be impossible for me to serve him in that way; but if he would bring his dentist to see me, I would consult with him, making such suggestions and rendering such assistance as would enable him to make great improvements on the set he had. The next day he called on me, in company with his dentist, whom I found to be an Austrian, from Vienna—a gentleman of culture, with a medical education, but quite deficient, in some respects, as a practical dentist. He was very cordial, seemed glad to see me (having seen my name in connection with the Philadelphia Dental College) and was gratified for the advice and aid I gave him.

I made an appointment to meet the patient at his office, where I took the impression, and so directed the construction of new dentures that before I left the city the patient was in possession of dentures that he found to be a great improvement on the former. He seemed delighted, and subsequently called at my hotel to express his gratitude, and to prevail on me, if possible, to remain and practice my profession in Beyrut, engaging if I would do so, that I should have all the business I could desire. I was obliged to decline his urgent invitation, thanking him for his appreciation of American dentistry, stating that I had a practice and professional interest in my own country that I could not be persuaded to leave. In Zurich, Switzerland, I found that a young American dentist, Dr. Senel, was building a most successful and lucrative practice. I found another American dentist at Florence, Italy. At Rome I called on Dr. Buridge, an American dentist, who enjoyed the reputation of being a fine operator, and was patronized by the Roman nobility and foreign residents.

For many years Dr. Brewster was the representative of our profession in Paris, and was *immediately* successful. It is probably known to most of you that it was with him that Dr. T. W. Evans became associated in practice, on his going to Paris some years ago, and no doubt to this fact, in part, as well as to his professional skill and personal merit, Dr. Evans is indebted for his unprecedented success. Probably no professional man, surgeon, physician or dentist, ever received the patronage of so many crowned heads, princes, nobles and persons of distinction and great wealth, as the American dentist, Dr. Thos. W. Evans, of Paris. His fees have been fabulous, and the emoluments of his practice have been so large, that his present wealth is estimated by millions. During my visit to Paris, in 1867. Dr. Evans was very cordial, and extended to me many acts of professional courtesy and personal kindness, which I fully appreciated, and to which I take pleasure in bearing my greatful testimony at this time. Dr. Gage was another American dentist, at that time enjoying an enviable reputation and success in Paris. In consequence of bad health, he was obliged to give up his practice, which was worth some $20,000 a year. Dr. Duboucet, of our city, negotiated for his practice, and became his successor, retaining most of his patients. Europeans seem to possess stronger local attachments than our restless, migrating American people. The dentist who büys out the good-will and practice of a European dentist, will be very likely, in case he is competent and can be strongly recommended, to retain nearly all the patients of his predecessors. Patients do not become scattered as they do in American cities.

The time may have gone by when American dentists can find such openings for practice as were found by Brewster, Evans, Gage and some others. But thirteen years ago there were great inducements offered to American dentists to open offices for practice in various cities of Europe. And I have no doubt of their being many most desirable opportunities for them now, not only on the Continent, but even in England, inasmuch as students from all nationalities come to the dental colleges of the United States of America for their education. I think the presumption is fair, that it remains for the American dentistry to supply the needs of the world, as it regards dental practice, to a large extent.—*Trans. Odontological Society.*

